# Supplementary material for: Are carbon emissions trading and green financial instruments synergistic? -Comprehensive quantitative research based on content analysis
Source: PLoS One. 2024 Mar 7;19(3):e0298601. doi: 10.1371/journal.pone.0298601 (PMC10919601; doi:10.1371/journal.pone.0298601)
Supplement: S1 Data — (ZIP) [file pone.0298601.s001.zip › Data/地级市Z_F工作报告.zip!/░▓╗╒/╦▐╓▌╩╨/└┤╫╘╣1⁄2╓┌║┼í╛┬φ┐╦╩2╛▌═°í┐_2009.docx]

2009年2月21日宿州市第三届人民代表大会第一次会议

市长  吴旭军

现在，我代表市人民政府，向大会报告工作，请予审议，并请市政协委员和其他列席人员提出意见。

一、过去五年工作回顾

市二届人大一次会议以来的五年，是我市改革发展进程中不平凡的五年。这五年，是我市经济实力大幅提升、结构调整实现历史性突破的五年，是基础设施逐步完善、城乡面貌变化巨大的五年，是社会事业全面发展、人民群众得到更多实惠的五年，是广大干群观念不断更新、信心显著增强的五年。全市人民在中共宿州市委的坚强领导下，全面落实科学发展观，按照市第二次、第三次党代会的部署，牢牢把握发展机遇，积极克服种种困难，深入实施五大战略，圆满完成本届政府任期内的各项目标，在奋力崛起的征程上迈出了坚实步伐。

综合实力大幅跃升。五年间，全市生产总值连跨三个百亿元台阶，由219亿元增加到511亿元，年均增长12.5%。财政收入由11.7亿元增加到27.4亿元，剔除取消农业税因素，年均增长26.4%。固定资产投资由42亿元增加到211亿元，年均增长38.2%，其中工业投入达到85亿元，是2003年的8.5倍。三次产业比例调整到30∶33∶37，二产比重提高9.6个百分点，成为拉动经济增长的主动力。全力组织工业突破，实施工业三年倍增计划，规模以上企业达546家，净增361家，户均产值是五年前的3倍，产值过亿元企业达到81家。煤电能源、加工制造、农副产品深加工三个百亿元产业不断壮大，华电一期、界沟煤矿、海升果业、新锦丰、惠丰电源、中元化肥等一批项目建成投产，原煤产量达1200万吨，电力装机容量达160万千瓦。企业自主创新能力逐步增强，建成12家省级企业技术中心，皖王小麦粉等18种产品分获中国、安徽名牌产品称号。全民创业蓬勃兴起，新增私营企业4000户，非公有制经济比重超过50%。农业基础日益稳固，粮食生产连续三年创历史新高，总产量达到73亿斤。高效种植、规模养殖比重逐年提高。“252”强龙工程目标提前一年完成，市级以上农业产业化龙头企业达159家，净增123家，其中国家和省级27家，营销总收入160亿元，为2003年的20倍，农民专业合作组织达到678个。绿色长廊工程建设、农机化综合水平保持全省领先。整村推进扶贫开发成效明显，新农村建设“十镇百村”示范工程顺利实施。加强劳务输出服务，外出务工人员达130万人。服务业稳步发展，社会消费品零售总额由68亿元增加到141亿元，年均增长15.8%。光彩城大市场等一批专业市场建成运营，皇藏峪晋升为国家4A级景区。县域经济发展提速，生产总值、财政收入等主要指标增幅连续两年超过全市平均水平，在全省县域经济考核中均实现位次前移，萧县成为全国防腐蚀业第一县，埇桥、砀山分别跻身全国粮食生产先进县、全省农业产业化先进县行列，灵璧、泗县连续多年被授予全省畜牧养殖十强县。乡镇经济加快发展，财政收入超500万元乡镇达到20个。

改革开放成果丰硕。农业税全面取消，累计发放粮食、农资等各类惠农补贴17.8亿元。农村综合改革稳妥推进，乡村区划调整如期完成。289家国有集体企业完成改制，一批骨干企业通过重组改造焕发生机。财税、金融体制改革取得新进展，公共财政体制不断完善，非税收入收缴管理制度全面推行，徽商银行入驻，农村信用联社完成统一法人改造，泗县农村合作银行获准筹建，中小企业融资担保体系、城市投融资体系基本形成。深入实施东向发展战略，全面融入徐州经济圈，积极参与泛长三角区域分工合作，累计引进内资410亿元、外资2.2亿美元,年均分别增长43.6%和31%。会展经济初具雏形，成功举办多届中国宿州灵璧石文化节、中国宿州黄淮海地区农资博览会、中国果蔬加工砀山论坛等节会，宿州知名度和影响力明显提升。宿州经济开发区基础设施日趋完善，实际引进资金、工业增加值、财政收入年均增长64%、149%、82%；县级开发区建设加快，完成基础设施投入6.6亿元，累计招商引资近30亿元；砀城、周寨、黄圩、朱仙庄、尤集成为全省第一批产业集群专业镇。大力发展外经外贸，进出口总额达2.6亿美元，年均增长30.4%。

城乡面貌日新月异。中心城市辐射带动能力显著增强，市区建成区面积由五年前的31平方公里扩大到50平方公里，人口由34万增加到50万。城乡规划体系逐步完善，城市总体规划等200项各类规划相继完成。市政设施建设步伐加快，累计完成投入30亿元，迎宾大道等40余条道路建成通车，沱河景观带等一批公园、广场建成启用，污水处理厂、垃圾处理厂投入运行。“三城同创”成效初显，荣获全省创建文明城市工作先进市、省级园林城市称号，绿化覆盖率提高10个百分点。汴河新区建设初具雏形，人气商机日益凸显。城市综合开发、旧城改造、配套设施建设步伐加快，累计竣工房地产面积420万平方米。建立市区联动的城管新机制，初步形成“两级政府、三级管理、四级网络”的格局。今天的宿州，已发展成为初具规模的中等城市。县城建设步伐加快，萧县、灵璧调整县城总体规划，泗县县城建设率先突破，砀山政务新区、萧县凤山新区、灵璧滨河新区建设顺利推进，城镇化率五年提高10个百分点。全力加快重大基础设施建设，京沪高铁、泗许高速、祁县船闸等项目陆续开工；奎濉河综合治理、沱河城区段治理、新汴河团结闸除险加固等工程全面竣工；一批输变电工程建成运行，城乡用电实现同网同价。县乡公路通达工程加快推进，建成“村村通”公路3593公里、广播电视“村村通”工程124处，新建农村沼气2万户、农家店1697个，解决43.7万农村人口饮水安全问题，砀山被列为全国农村饮水安全工程示范县。“数字宿州”框架基本建立，宽带数据网覆盖到乡镇，移动通信、光纤通信覆盖到村。

社会事业全面进步。实施免费义务教育，受益学生160多万，为197万名农村学生和城市贫困家庭学生免费提供教科书，对3.5万名寄宿学生补助生活费，消除农村中小学D级危房67万平方米；中小学布局调整稳妥推进，教育资源配置进一步优化；高考本科上线人数连续多年居全省前列；民办教育机构增加到272所；宿州学院、宿州职业技术学院成功组建，宿州卫校实现整体搬迁。文化事业繁荣发展，文化场馆建设步伐加快，泗州戏等21个项目入选国家、省级非物质文化遗产名录，文化产业列入国民经济发展规划。群众性精神文明创建活动深入开展，“和睦和衷、尚贤尚信、大志大气、决战决胜”的宿州精神深入人心。公共卫生体系不断健全，120急救中心、传染病院区和各级疾病控制中心建成；新型农村合作医疗制度覆盖全市，参合率达85.8%，新建改建乡镇卫生院103所、村卫生室367个、社区卫生服务机构62个；有效防治手足口病等重大疫病，妥善处置问题奶粉事件。荣获全国无偿献血先进市称号。竞技体育水平不断提高，我市健儿在奥运会、残奥会上勇夺5枚金牌、1枚银牌和1枚铜牌。计划生育基础工作不断夯实，低生育水平持续稳定，出生人口性别比五年下降8个点。节能减排取得阶段性成效，土地利用集约化逐年增强。“平安宿州”建设扎实推进，应急管理体系逐步完善，大信访工作格局初步形成，安全生产形势总体平稳，社会大局保持稳定。“四五”普法顺利完成，“五五”普法启动实施。支持驻宿部队建设，国防动员、民兵预备役和人防建设进一步加强，连续五次荣获“全国双拥模范城”称号。老龄、妇女、儿童及残疾人合法权益得到保障。民族宗教、人事编制、外事侨务、旅游、新闻出版、防震减灾、气象、物价、统计、档案、外宣、对台、地方志等工作均取得新进展。

改善民生成效斐然。城乡居民收入水平不断提高，城镇居民人均可支配收入由6196元增加到11899元，年均增长13.9%；农民人均纯收入由1707元增加到3671元，年均增长16.5%；居民储蓄存款余额达到345亿元，年均增长18.4%。强化就业服务和再就业帮扶，新增城镇就业岗位12.1万个，1.4万困难群体人员实现稳定就业，城镇登记失业率稳控在4.3%以内。社会保障体系不断完善，社会保险五大险种初步建立，运行质量明显提高。最低生活保障制度覆盖城乡，被征地农民纳入社会保障体系，5738户居民享受城市廉租房补贴。特别是近两年，我们先后实施12项和18项民生工程，并结合群众需求建立项目补充机制，累计投入19.8亿元，惠及400多万城乡居民。3.3万名农村“五保户”落实供养标准，1.2万个农村计划生育家庭享受奖扶资金，37.8万名城乡特困群众得到医疗救助，1221名重大传染病人得到医疗救治和生活救助，2.4万名中职学校贫困生得到资助，为400名白内障患者免费实施复明手术。公厕建设、小街小巷改造列入民生工程，受到人民群众的普遍欢迎。

民主法制和政府自身建设不断加强。围绕建设学习型、服务型和效能型政府，修订《宿州市人民政府工作规则》，进一步规范了政府工作程序。自觉接受人大法律监督、政协民主监督和社会舆论监督，注意听取各方面的意见和建议，按期办结人大议案15件、代表建议427件、政协委员提案1052件。开通市民论坛、行风热线，搭建民声热线和基层信访两个平台，建立民生问题服务承诺制度。实施政府信息公开，建立重大决策网上征求意见制度。先后开展纪律作风集中整顿、机关效能建设、“两风”建设和“四民”活动，解决了一大批影响发展的突出矛盾和问题。认真贯彻落实《行政许可法》，成立市政府法律顾问组，开展行政审批清理工作，取消、调整行政审批项目508项。加强行政监察和审计监督，落实党风廉政建设责任制，加大对商业贿赂和损害群众利益等不正之风的治理力度，政风建设取得新成效。

过去的一年，是本届政府任期最后一年。这一年，我们经历了一些始料未及的重大挑战。在考验面前，我们众志成城，战胜了罕见的冰冻灾害；沉着应对，及时遏制了突如其来的手足口病疫情；审时度势，经受了国际金融危机的冲击，坚持一手抓“项目建设年”活动促经济发展，一手抓“四民”活动保和谐稳定，主要经济指标均达到近几年最好水平。全市生产总值增长14.1%，财政收入增长28.6%，均高于全省平均水平；规模以上工业增加值增长35.7%，增幅居全省第二；固定资产投资增长65.9%，增幅居全省第一；社会消费品零售总额增长23.1%；城镇居民人均可支配收入增长12.7%；农民人均纯收入增长19.5%。

各位代表，实践孕育真知，历史昭示未来。过去五年，我们在困难中奋起，在开拓中前行，积累了非常宝贵的经验。实践启示我们，加速宿州崛起，必须坚持解放思想不动摇。五年来，我们把上级要求与宿州实际紧密结合起来，把握新一轮经济增长周期的有利时机，抢抓中部崛起和省委、省政府加快皖北地区发展的大好机遇，不断创新发展理念，着力突破瓶颈、消除障碍、化解难题，探索出一条符合市情的发展道路。必须坚持发展至上不动摇。五年来，我们紧紧围绕“两个跃升、一个崛起”的奋斗目标，确立了以农业为基础，以工业化、城市化为核心，以结构调整为主线的发展思路，作出了实施“6＋3”工程、工业三年倍增、举全市之力建设开发区等一系列重大决策，使发展方向更加明确，发展重点更加清晰。必须坚持改革开放不动摇。五年来，我们着力冲破体制机制上的束缚，坚持大开放主战略，把招商引资作为经济工作的生命线，以融入徐州经济圈为跳板，瞄准苏浙沪，对接长三角，在工业发展、城市建设、社会事业等领域广泛吸引国内外投资者，为快速发展提供了有力支撑。必须坚持改善民生不动摇。五年来，我们始终坚持以人为本，把保障和改善民生作为一切工作的出发点和落脚点，下大气力推进社会建设和改善民生，下大决心解决人民群众最关心、最直接、最现实的利益问题，让改革发展的成果惠及全市人民。

各位代表，回顾五年来的发展历程，我们倍感成绩来之不易，深知探索之艰辛。五年来，我们之所以能够战胜一场又一场自然灾害，之所以能够妥善应对一次又一次宏观形势变化，之所以能够取得一个又一个进步，靠的是全面贯彻落实科学发展观，靠的是省委、省政府和市委的正确领导，靠的是全市人民同心同德、团结奋斗。在此，我谨代表市人民政府，向全市广大干部群众，向各民主党派、工商联和人民团体，向驻宿解放军、武警官兵和政法干警，致以崇高的敬意！向关心、支持宿州发展的中央、省驻宿单位和海内外友好人士，向在我市创业的投资者、建设者，表示衷心的感谢！

各位代表，回顾五年来的发展历程，我们还必须清醒地看到，尽管宿州发展已迈入加速崛起的新阶段，但在省内人口总量靠前、经济总量居中、人均指标靠后的状况尚未根本改变；结构层次不高、新兴产业发育不足、过度依赖资源性产业的格局尚未根本改变；城乡居民收入与全国、全省平均水平相比仍有较大差距；影响社会稳定的因素依然不少；各级政府引领发展的能力有待提高。对此，我们一定高度重视，认真解决，决不辜负全市人民的期望与重托！

二、今后五年的形势与任务

今后五年，是我市深入贯彻党的十七大和十七届三中全会精神，衔接两个五年发展规划，承前启后、继往开来的关键时期。纵观全局，这一时期，机遇前所未有，挑战也前所未有，总体上机遇大于挑战。从外部环境看，尽管当前受到全球金融危机的冲击，但经济全球化深入发展的大趋势没有改变，我国经济发展的基本面没有改变，发展的重要战略机遇期仍然存在，中部崛起战略和省委、省政府加快皖北发展的政策将会进一步深化，都为我市改善基础设施，调整产业结构，增强发展后劲带来了有利条件。从发展阶段看，经过多年积累，我市二产在三次产业结构中的比重超过一产，人均生产总值突破1000美元，已经步入工业化、城镇化加速发展的新阶段，经济增长的稳定性、协调性明显增强，再加上全民创业、招商引资积聚的能量加速释放，区位交通、资源环境优势进一步凸显，广大干部应对复杂局面、解决实际问题的能力不断提高，人民群众谋发展、盼富裕的愿望更加强烈，宿州既有跨越发展的需要，更有跨越发展的可能。在这个关键阶段，我们必须抓住一切可用机遇，利用一切有利条件，调动一切积极因素，不动摇，不懈怠，不折腾，努力推动宿州在新的起点上实现更好更快发展。

市委三届五次全会立足新的起点，规划了未来五年宿州发展的宏伟蓝图，提出用五年或更长一段时间，力争实现“双千亿、双翻番”的奋斗目标，为新一届政府指明了前进方向。未来五年，我们将高举中国特色社会主义伟大旗帜，以邓小平理论和“三个代表”重要思想为指导，深入贯彻落实科学发展观，按照市第三次党代会和市委三届五次全会的战略部署，围绕“全面追赶、重点突破、局部超越”的总体要求，坚持以工业化为核心，充分发挥煤炭、农产品和人力资源三大优势，大力实施工业扩张、城镇扩容、农业提升三大战略，加快工业化、城镇化和农业现代化进程，协调推进经济、文化、社会和生态建设，努力构建产业发达、城乡优美、社会和谐、民生殷实的新宿州，全面实现“两个跃升、一个崛起”的奋斗目标。

今后五年的发展目标是：

——综合实力显著增强。经济总量上台阶进位次，人均上水平，主要经济指标增速高于全省，进一步缩小相对差距，提高对全省经济发展的贡献率。力争到2013年，全市生产总值超过1000亿元，年均增长13%以上，在全省前移1到2个位次；二三产业占地区生产总值的比重超过80%；财政收入保持同步增长，占GDP比重逐年提高；工业固定资产投资年均增长30%以上；城镇居民人均可支配收入、农民人均纯收入分别年均增长10%以上；县域经济总量、工业增加值、财政收入占全市总量的比重明显提升，在全省综合排名中位次明显前移，综合实力跻身全省中上游水平。

——工业支撑能力显著提升。突出工业主导地位，坚定不移地推进工业扩张战略，加大决战工业力度，提升产业结构层次，以工业率先突破带动发展整体跨越。立足资源优势和产业基础，坚持发展煤电能源、加工制造、农副产品深加工三大产业基地，打造城市和乡村两大工业平台，通过规划引导、政策扶持，力争到2013年，规模以上工业增加值达到350亿元，年均增长25%以上，规模以上企业超过1000户，组建一批充满活力的企业集团，形成一批特色明显的产业集群，初步构建具有较强竞争力的现代工业体系。着眼服务业和加工制造业的相互促进，适应产业结构和消费结构升级趋势，优先发展生产性服务业，着力提升生活性服务业，建设一批高质量的物流园区，培育一批工业原料、生产资料市场，逐年提高服务业对经济发展的贡献率。

——农业基础地位显著巩固。坚持以工业化致富农民、城镇化带动农村、产业化提升农业，大力实施农业提升战略，积极构建“四大产业主导、三位一体发展、八个体系支撑”的现代农业产业体系。大幅度增加对农业的投入，鼓励和引导社会各方面资金投向农村，切实加强农业基础建设，突出抓好重点水利工程和农田基本建设，改善农业生产条件，实施粮食增产规划。加快农业产业结构调整，大力发展现代农业，提高集约化、组织化、规模化程度。实施农业产业化提升行动，省级以上龙头企业达到200家，营销收入过亿元企业超过60家，农民专业合作组织突破1000个。把社会主义新农村建设作为农村改革发展的战略任务，坚持典型示范和整体推进相结合，加快农村公共设施建设和社会事业发展。认真落实各项惠农政策，采取综合措施，促进农民增收。

——城乡面貌显著改观。坚持中心城市与县域经济互动发展，城镇化与新农村建设同步推进，促进城乡规划、产业布局、基础设施、公共服务和社会管理一体化。立足皖东北中心城市和商贸中心的发展定位，修编市域城镇体系规划。继续做大中心城市，加快基础设施建设，完善城市配套服务功能，提升城市品位，增强辐射带动力，力争到2013年，市区建成区面积超过70平方公里，人口超过70万人；以推进基础设施建设为重点，做大县城和中心镇规模，城镇化率提高到45%左右，初步构建以宿城为中心、各县城为副中心、重点中心镇组成的城镇体系。加快推进高速铁路、高速公路和干线公路建设，构筑中心城市到各县的快速通道，打造更加顺畅便捷的城乡交通体系。

——改革开放步伐显著加快。坚定不移地推动改革，破除影响发展的各种障碍，努力在更大范围、更高层面集聚要素。加快政府职能转变，深化行政管理体制改革，着力提升政府服务发展的能力。加快企业改革步伐，深入推进全民创业行动，大力发展非公有制经济。深化金融改革，加快地方金融机构组建，培育做大金融市场。积极培育产权、土地、技术等要素市场，规范发展行业协会和中介组织。推进“诚信宿州”建设，构建统一开放、竞争有序的现代市场体系。加速发展开放型经济，扩大利用外资的规模，力争累计到位内资突破1000亿元、外资6亿美元，年均分别增长30%、15%。

——经济发展方式显著转变。立足科学发展，突出好中求快、优中求进，形成有利于可持续发展的经济发展方式。大力发展循环经济，全面加强节能、节地、节水、节材和资源综合利用工作，推行清洁生产，组织实施一批循环经济项目，培育一批循环经济园区和企业。牢固树立生态文明观念，推进生态宿州建设，建设保护好生态功能区，全面治理点源、面源污染，形成节约能源资源和保护生态环境的产业结构、增长方式、消费模式，确保单位生产总值能耗和主要污染物排放总量控制在省下达的指标以内。

——人民群众生活水平显著提高。把发展经济与造福百姓结合起来，加快实施以民生工程为重点的社会建设，使人民群众学有所教、劳有所得、病有所医、老有所养、住有所居。优先发展教育事业，优化教育资源布局，促进各类教育均衡公平发展。建立完善覆盖城乡居民的公共卫生服务体系、医疗服务体系、医疗保障体系和药品供应保障体系，力争城镇人人享有医疗保险，农民人人参加新型农村合作医疗。发挥地方文化特色，打造文化产业，培育文化品牌。加快科技创新体系建设，增强自主创新能力。推进各项社会事业协调发展。完善住房供应体系，解决中低收入家庭住房困难。加快建设覆盖城乡居民的社会保障体系，促进城乡居民充分就业。大力建设平安宿州，增强人民群众的安全感。

各位代表，抚今追昔，意在登高望远；知往鉴今，重在开创未来。今天的宿州，正站在奋力起跳的历史节点上，正处在加速跨越的新阶段。我们要以只争朝夕、时不我待的强烈意识，敢闯敢试、锐意进取的奋斗品质，埋头苦干、不尚空谈的实干精神，不为任何风险所惧，不为任何干扰所惑，合力攻坚，破浪前进，构筑发展新平台，开创发展新局面，塑造宿州新形象，让宿州这座皖北名城焕发出更加蓬勃的生机与活力！

三、2009年主要工作安排

今年是宿州撤地建市10周年，是实现“双千亿、双翻番”目标的起步之年，也是新一届政府的开局之年。做好今年的各项工作，对于有效应对金融危机冲击，保持我市来之不易的良好发展势头至关重要。我们要深入贯彻落实科学发展观，把握国际国内发展环境新变化，把保持经济平稳较快增长作为首要任务，深入实施三大战略，全力以赴扩大投资，着力推进改革开放，着力优化经济结构和提高经济发展质量，着力加强节能减排和环境保护，着力改善民生和促进社会和谐，为今后五年的发展开好局、起好步。

综合考虑各方面因素，今年经济社会发展的主要预期目标是：全市生产总值增长12%以上；财政收入保持同步增长；规模以上工业增加值增长25%以上；固定资产投资和招商到位资金均增长30%；全社会消费品零售总额增长15%；城镇居民人均可支配收入和农民人均纯收入均增长10%；人口出生率控制在13‰以内；单位GDP能耗及主要污染物减排量达到省控目标。确定上述目标，主要是考虑到，今年经济发展中还存在诸多不确定因素，尤其是金融危机对实体经济的影响还在进一步加深，对我市影响的滞后性、传导效应尚未完全显现，这个目标是实事求是、相对稳妥的。但实际工作中，我们将坚持能快则快的原则，坚定信心，知难而进，力求发展得更快、完成得更好。

今年将着重抓好八个方面的工作：

（一）扩大有效投入，保持经济平稳较快增长。把扩大投资作为保增长的主攻方向，紧紧抓住国家增加投资规模的战略机遇，继续深化“项目建设年”活动，以项目大建设推进宿州大发展。

全面提升“6＋3”工程。准确把握国家宏观政策导向，建立专门班子，落实专项经费，统筹推进项目筛选、储备、谋划、申报和实施工作。完善项目管理联动机制，落实规划、土地、环评等建设条件，尽早开工、竣工一批项目。深化项目调度机制，实施报告、协商、督查和考核四项制度。充分利用积极的财政政策和适度宽松的货币政策，认真实施好国债项目，带动社会投资，加速基础设施、产业体系和公共服务设施建设，逐步形成政府投资引导、社会资本和金融资金跟进的格局。

加快基础设施建设，重点抓好“三铁三高三路两河”。加快京沪高铁建设，争取宿淮铁路、郑徐客运专线早日开工；加快泗许高速公路建设，争取徐明、济祁高速公路早日开工；加快303省道改建，做好市区到蒿沟高铁连接线、206国道改线项目规划设计工作；加快浍河祁县船闸建设，做好新汴河综合治理项目申报工作，着力提升宿州区域交通优势。加快建设220千伏宿南变等一批输变电工程，着力提升供电保障能力。完善信息化基础设施和服务体系，大力支持第三代移动通信网建设。

加速构建煤电产业基地，重点抓好“五煤五电”。力争钱营孜煤矿建成投产，金黄庄和金狮矿业完成年度投资计划，骑路孙煤矿开工建设，张大屯煤矿早日核准；力争国电“大代小”热电联产开工建设，华电二期、钱营孜煤矸石电厂早日核准，加快皖能宿州发电、大唐祁东电厂前期工作。

（二）扩张工业总量，强化工业主导作用。把工业发展作为保增长的着力点，坚持一手保企业运转，一手抓产业培育，进一步提升工业对经济发展的带动力。

企业是发展之基，必须采取更直接、更有力、更管用的办法保企业。充分发挥财政资金的杠杆作用，建立完善担保基金和风险补偿基金，利用好财政贴息、项目补助、工业发展专项资金，引导企业加快结构调整、产业升级、技术改造。多形式、多渠道推动银企合作，加大对有市场、有效益企业的资金扶持。完善商业银行考核体系，引导金融机构扩大对企业的有效信贷投放，确保信贷增幅高于全省平均水平。利用金融危机形成的倒逼机制，指导企业练好内功，拓宽市场空间，在应对危机中转型升级，在市场竞争中发展壮大。加大对企业的帮扶力度，抓好百户亿元以上企业运行调度。深入开展“察企业所想、解企业所忧、为企业服务、促企业发展”为主题的优化发展环境活动，切实规范执法行为，全面清理各项涉企费用，采取取消、下调、暂缓征收等办法，最大限度地减轻企业负担。

加快产业结构优化升级。围绕三个百亿元产业基地建设，提升主导产业发展规划和实施方案，打造一批亿元项目，培植一批十亿元企业，形成一批销售收入超五十亿元的产业集群。深化企业内涵改造和外延扩张，重点抓好30个投资3000万元以上的技改项目。加快福润食品、汇源果汁、中元化肥、天瑞水泥、雪龙化纤、奥吉斯电器、惠丰铸造、中腾煤焦电、旗杆楼铁矿等项目建设，力争工业投资增幅高于全市固定资产投资增幅。发挥企业自主创新的主体作用，建立多种形式的产学研合作，促进各类创新要素向企业集聚，增强企业核心竞争力。

认真落实节能降耗和减排目标责任制，严把环境准入关。突出抓好工业、交通、建筑三大领域节能，提高污水、垃圾处理能力，加大污染治理，防止重特大污染事故，抓好40户重点耗能企业的节能降耗，确保完成节能减排任务。

（三）加速城镇扩容，增强区域经济承载功能。把加速城镇化进程作为应对当前经济形势的战略举措，掀起新一轮城乡建设热潮，着力提升城镇综合承载力。

发挥中心城市在城镇体系中的龙头作用。调整城市总体规划，修编土地利用总体规划，抓好16个专项和详细规划编制，实现宿城近期建设控制性详规全覆盖。继续加快城市基础设施建设，突出道路连网、环境提升、公共服务等重点，投资16亿元，实施55项重点工程，其中道路工程14项、园林景观及城市出入口节点建设工程5项、公共公用设施和民生工程30项、廉租房及拆迁安置房建设项目6项，进一步拉开城市框架，完善城市功能，改善人居环境。继续推进三区联动发展，提升汴河新区南片商业、服务功能，加快学校、市民活动中心等公共设施建设；完善西南片区产业功能，重点建设一批专业市场；加快道东片区改造步伐，改善市民居住条件。巩固省级园林城市创建成果，加快省级文明城市、卫生城市创建步伐。强化城市管理，开展综合执法推进年和城市网格化服务推进年活动，实现城市管理服务常态化、长效化。

增强县城和小城镇辐射带动能力。支持四个县城以新区开发为牵动，加速与中心城区的对接融合，提升副中心的地位和功能。加快砀山、泗县县城总体规划修编调整，促进泗县争创省级文明县城。择优培育一批重点中心镇及经济强镇，加强基础设施配套建设，集约使用土地资源，引导乡镇企业向工业功能区集中，镇村居民向小城镇集中，使县城和小城镇成为县域经济的增长极、工业的集中区、人口的集聚地。以科学规划引领社会主义新农村建设。

（四）加强“三农”工作，振兴县域经济。把农业作为安天下、稳民心的战略产业，围绕稳粮、增收、强基础、重民生，加大投入力度，千方百计促进农民收入持续增长。

着力抓好农业基础设施建设，完成110万亩易涝农田综合治理，扩大土地整治和中低产田改造，实施重点平原洼地治理、宿县闸加固、岳庄坝水库续建及小水库加固等工程，改善农业生产条件。积极推进村庄整治和宅基地置换，重点办好“水、电、路、气、房”五件实事，加快电气化村镇建设，加强“村村通”道路延伸工程建设，逐步消除乡村断头路，再解决28万人的饮水安全问题。实施农业产业化提升行动，落实扶持农业产业化经营的各项政策，培育壮大龙头企业，加快发展农民专业合作组织，提高农业和农村经济组织化程度。继续实施小麦高产攻关、玉米振兴计划行动，确保粮食增产增收。进一步优化农业结构和区域布局，建设优势农产品基地，力争畜牧业规模养殖比重提高5个百分点。推行农业标准化管理，严格农产品质量安全全程监控。加快建立农业社会化服务体系，健全农业技术推广、动植物疫病防控等公共服务机构。高度重视农民工就业问题，加强面向农民工的技能培训和信息服务，制定农民工就业扶持政策，鼓励中小企业、重大基础设施项目建设更多使用农民工，加快建设农民工返乡创业园，鼓励有条件的农民工返乡创业。认真落实增加农业补贴、提高粮食最低收购价等政策，让农民真正得到实惠。

县域经济既是我们的差距所在，也是崛起的潜力所在，必须把县域经济放在更加突出的战略位置，加快振兴步伐，促进县域经济发展提质增量。全面落实省委、省政府加快皖北和沿淮部分市县发展政策，在产业布局、项目安排、建设用地等方面向县区倾斜，增强县区自我发展能力。坚持分类指导，进一步完善县域经济考核办法，强化对县域经济的支持与服务，支持埇桥区发展加工制造、城市服务业，提高经济首位度；推进砀山、萧县分别向全国水果加工第一大县、粮食加工第一大县迈进；促进灵璧、泗县在农副产品深加工和劳动密集型加工配套产业方面有更大突破，引领各地走特色发展之路。壮大乡镇经济规模，增加乡镇财税收入，培植一批工业增加值过亿元、财政收入过千万元的乡镇。

（五）深化各项改革，提高对外开放水平。把深化改革、扩大开放作为保增长的强大动力，大力革除影响科学发展的体制机制障碍。

深化重点领域和关键环节改革。加速国有集体企业改革改制步伐，加快重点企业联合重组、嫁接改造。深化财税管理体制改革，调整财政支出结构，加大财税对经济发展的支持力度。大力发展多种所有制、多种经营形式的银行机构和非银行金融组织，支持金融机构加大服务网点建设，推进农村信用社改制为农村银行，加快小额贷款公司和村镇银行组建步伐，争取实现小额贷款公司县域全覆盖。推进信用体系建设，畅通政银企沟通渠道，改善金融生态环境。适时启动市、县区政府机构改革，深化事业单位人事制度改革，建立健全事业单位岗位设置管理制度，逐步实现政事分开、事企分开和管办分离。落实各项政策措施，完善有利于非公有制经济发展的体制机制，鼓励和促进非公有资本进入公用事业、基础设施、社会事业等领域。继续深化农村综合改革，坚持依法自愿有偿原则，促进土地承包经营权流转，加快集体林权和征地制度改革步伐，增强农业发展活力。

继续扩大对外开放。注重提升引资质量，加大先进制造业、高新技术等产业招商力度，积极引进知名大企业和战略投资者，推进企业扩张、产业结构升级和新兴产业培育。强化专业招商分局、产业招商中心、小分队建设，提高招商人员实战技能。加强招商引资目标考核，建立健全良好的运作机制。广泛参与我省融入泛长三角区域有关活动，有针对性组织主题招商活动，办好第五届黄淮海农资博览会和第四届国际腐蚀控制大会，提高节会招商成效。继续加快各类园区建设，支持宿州经济开发区强化基础设施建设，打造核心企业、核心产业、核心竞争力，积极争创国家级开发区；充分利用省扶持政策，突出比较优势，培育特色产业，把县区开发区打造成工业发展的主阵地；加快发展乡镇工业集中区，选准主导产业，实现错位发展，建成各具特色的承接平台。

（六）发展壮大服务业，拉动消费需求。把推进服务业发展作为扩大内需的重要切入点，增强消费对经济增长的拉动作用。围绕建设皖东北商贸中心，积极引进家乐福、大润发、乐购、苏宁电器等知名流通企业，运用新型商业业态提升商业档次；加快中央大厦、皖煤五星级大酒店等商业基础设施建设，打造一批特色商业街，构建“井”字型中心商圈。大力发展奇石、蔬菜批发、农机农资等专业市场，培育一批县域特色市场，优化消费硬件环境。推进蒿沟物流园和粮食物流园建设，发展现代物流业。培育引进一批规模较大、实力雄厚的生产性服务业，重点支持信托、保险、证券、期货等非银行金融机构发展。落实房地产业相关政策，规范、引导房地产市场有序发展。完善物业、社区、保洁等面向民生的生活性服务业。充分利用丰富的人文、自然资源，加快旅游基础设施建设，打造三大旅游板块。继续推进“万村千乡”市场工程，组织开展家电、农机下乡等活动，新建、改造一批农贸市场、农家店，搞活农产品流通，加快农村消费结构升级。

（七）坚持协调发展，推进各项社会事业。把满足人民群众日益增长的公共服务需求作为扩大内需的重要方向。优先发展教育事业，巩固九年义务教育，开展农村中小学校标准化建设年活动，继续优化中小学校布局，强化薄弱学校建设。加强师资队伍建设，完成新一轮中小学教职工编制核定工作。扩大优质高中规模，提高高等教育办学层次。大力发展职业教育，启动职教园区建设，组建宿州技师学院，推动宿州卫校升格。完善公共医疗卫生服务体系，加快市立医院、皖煤医院综合楼建设，逐步改善市中医院、县级医院、妇幼保健院（所）、乡镇卫生院、村卫生室、社区卫生服务机构等医疗服务条件，提升医疗服务水平。巩固发展城镇居民基本医疗保险和新型农村合作医疗制度，不断提高城乡居民的受益面和受惠水平。大力发展文化事业，传承传统文化艺术，加强文物、非物质文化遗产保护。推进公共文化服务工程建设，构建覆盖全社会的公共文化服务体系。加快区域性特色文化产业群建设，以文化产业大发展促进文化事业大繁荣。开展全民健身活动，提高竞技体育水平，积极备战全运会。

提高人口素质，依法治理出生人口性别比过高问题，扩大计划生育奖励扶助范围，加强计生技术服务机构和队伍建设。深入开展双拥活动，增强全民国防意识，巩固发展军政军民团结。依法加强民族宗教事务管理。深入开展质量和安全年活动，加强食品药品安全监管，进一步规范市场秩序。继续搞好全国第二次经济普查。充分发挥工会、共青团、妇联等群团组织的桥梁纽带作用。关心支持老龄、残疾人、红十字会工作，重视外事、侨务、对台、档案、地方志、气象和防震减灾工作。深入开展“五五”普法工作。深化大调解机制、大防控体系和基层基础建设，完善信访三级平台建设，依法妥善处理群众反映的突出问题。建立健全应急管理体系，落实安全生产责任制，防止重特大事故发生。加强社会治安综合治理，严厉打击各类犯罪活动，建设平安宿州，保障人民群众安居乐业。

（八）不断改善民生，让改革发展成果惠及全市人民。把改善民生作为保增长的出发点和落脚点，不断提高人民群众生活和谐度、满意度。巩固提升民生工程，将18项民生工程扩充至28项民生工程，新增农民体育健康工程、政策性农业保险试点、农村沼气建设工程、提高妇女儿童健康水平、乡镇综合文化站建设、“农家书屋”工程、病险水库除险加固工程等11个项目，努力让人民群众得到更多的实惠。继续加大财政投入，建立稳定多元的筹资机制，完善项目资金保障监管，及时足额落实配套资金。各级领导干部要牢固树立过紧日子的思想，带头厉行节约，严格实行公务购车、会议、党政机关出国经费零增长制度，把更多的资金放在保增长、保企业、保民生上。巩固“四民”活动成果，完善民声热线系统平台，全面清理涉及民生的价格收费，严厉查处各种乱收费。实施更加积极的就业政策，落实税收优惠、小额担保贷款等政策，鼓励和扶持更多劳动者自谋职业、自主创业。稳定现有就业岗位，引导企业履行社会责任。开展就业服务系列活动，鼓励大学生到基层、农村、中小企业就业。稳步提高城市低保标准，扩大农村低保对象范围。拓展城镇职工基本养老覆盖面，启动新型农村养老保险制度试点，全面落实被征地农民养老保险政策,提高城乡居民应对风险的能力。新建、改建一批五保供养服务机构，努力提高农村五保集中供养率。逐步扩大城乡特困群众医疗救助范围，完善临时救助管理和流浪乞讨人员救助制度，发展社会福利、优抚安置、慈善和残疾人事业，让更多的困难群众受益。加大社会保障性住房建设力度，加快城镇棚户区、危旧房改造，做好低收入家庭和低保家庭住房保障工作。

四、加强政府自身建设

实现未来的宏伟蓝图，完成今年的目标任务，需要全市上下的共同努力，需要政府自身的积极作为。我们必须全面落实科学发展观，加强自身建设，努力建设人民放心满意的政府。

（一）坚持解放思想，着力构建开放型政府。深入开展学习实践科学发展观活动，顺应发展大势，应对复杂局势，换脑筋、转观念、拓思路，革除不利于科学发展的思维定势和行为习惯，突破不利于加速崛起的条条框框和陈规陋习，以更广的眼界审视发展，以更大的气魄加快发展，以更实的举措推动发展。正确把握经济规律，在积极应对宏观调控中抢抓机遇，努力增强工作的创造性、预见性和主动性。强力推进工作创新，只要有利于宿州发展的事情，就大胆去试、大胆去闯、大胆去干，宽容探索中的失误，允许创新中的曲折，把方方面面的创造活力充分激发出来，形成跨越发展的强大合力。

（二）坚持提升效能，着力构建服务型政府。进一步转变政府职能，坚持高效运转，减少和规范行政审批，优化服务流程，简化办事环节。建立政务公开长效机制，推进政府信息公开。加强政府信息化建设，精文简会，降低行政成本。加强行政效能监察，推行限时办结制，落实责任追究制，坚决制止不负责任、推诿扯皮的现象，确保政令畅通。大兴调查研究之风，真诚倾听群众呼声，真实反映群众愿望，真情关心群众疾苦，多为群众办好事、办实事。

（三）坚持依法行政，着力构建法治型政府。加快依法治市进程，严格依照法定权限和程序行使权力、履行职责，做到有法必依、执法必严、违法必究。自觉接受人大及其常委会的法律监督和政协的民主监督，认真听取各民主党派、工商联、无党派人士及人民团体的意见和建议，积极办理人大代表议案、建议和政协委员提案。做好行政复议和法律援助工作。强化对权力的制约，特别要加强对领导干部、人财物管理使用、关键岗位的监督和制约，防止权力失控、决策失误、行为失范。完善政府决策机制，提高决策科学化、民主化、规范化水平。

（四）坚持廉洁从政，着力构建廉政型政府。大力弘扬廉政风尚，坚持标本兼治、综合治理、惩防并举、注重预防的方针，扎实推进反腐倡廉制度建设。认真落实党风廉政建设责任制和领导干部廉洁自律的各项规定，加强领导干部经济责任审计，拓展从源头上防治腐败的工作领域。深入开展行风政风评议活动，着力纠正行业和部门不正之风，查处违纪违法案件，严肃整治损害群众利益和破坏投资环境的行为。加强对公务员的教育、管理和监督，以廉洁奉公的政府形象取信于民。

各位代表！时代赋予重托，团结凝聚力量，奋斗铸就辉煌。让我们更加紧密地团结在以胡锦涛同志为总书记的党中央周围，高举中国特色社会主义伟大旗帜，深入贯彻落实科学发展观，在省委、省政府和市委的坚强领导下，解放思想，抢抓机遇，锐意进取，埋头苦干，以宿州加速崛起的新成就，向新中国成立60周年献礼！
